# *In vivo* Non-invasive Imaging of Radio-Labeled Exosome-Mimetics Derived From Red Blood Cells in Mice

**DOI:** 10.3389/fphar.2018.00817

**Published:** 2018-07-30

**Authors:** Prakash Gangadaran, Chae Moon Hong, Ji Min Oh, Ramya Lakshmi Rajendran, Senthilkumar Kalimuthu, Seung Hyun Son, Arunnehru Gopal, Liya Zhu, Se Hwan Baek, Shin Young Jeong, Sang-Woo Lee, Jaetae Lee, Byeong-Cheol Ahn

**Affiliations:** ^1^Department of Nuclear Medicine, School of Medicine, Kyungpook National University, Daegu, South Korea; ^2^Department of Nuclear Medicine, Kyungpook National University Hospital, Daegu, South Korea

**Keywords:** red blood cell, exosome-mimetic, non-invasive imaging, radiolabeling, drug delivery

## Abstract

Exosomes are natural nano-sized membrane vesicles that have garnered recent interest owing to their potential as drug delivery vehicles. Though exosomes are effective drug carriers, their production and *in vivo* biodistribution are still not completely elucidated. We analyzed the production of exosome mimetics (EMs) from red blood cells (RBCs) and the radio-labeling of the RBC-EMs for *in vivo* imaging. Engineered EMs from RBCs were produced in large-scale by a one-step extrusion method, and further purified by density-gradient centrifugation. RBC-EMs were labeled with technetium-99m (^99m^Tc). For non-invasive imaging, ^99m^Tc (free) or ^99m^Tc-RBC-EMs were injected in mice, and their biodistribution was analyzed by gamma camera imaging. Animals were sacrificed, and organs were collected for further biodistribution analysis. RBC-EMs have similar characteristics as the RBC exosomes but have a 130-fold higher production yield in terms of particle numbers. Radiochemical purity of ^99m^Tc-RBC-EMs was almost 100% till 2 h reduced to 97% at 3 h. Radio-labeling did not affect the size and morphology of RBC-EMs. In contrast to free ^99m^Tc, *in vivo* imaging of ^99m^Tc-RBC-EMs in mice showed higher uptake in the liver and spleen, and no uptake in the thyroid. *Ex vivo* imaging confirmed the *in vivo* findings. Furthermore, fluorescent imaging confirmed the nuclear imaging findings. Immunofluorescent imaging revealed that the hepatic uptake of RBC-EMs was significantly mediated by kupffer cells (resident hepatic macrophages). Our results demonstrate a simple yet large-scale production method for a novel type of RBC-EMs, which can be effectively labeled with ^99m^Tc, and feasibly monitored *in vivo* by nuclear imaging. The RBC-EMs may be used as *in vivo* drug delivery vehicles.

## Introduction

Exosomes are naturally occurring biological nanovesicles (30–200 nm) released by normal cells such as macrophages (Saari et al., 2015), mesenchymal stem cells (Gangadaran et al., 2017c; Rajendran et al., 2017), natural killer cells (Zhu et al., 2017, 2018), and red blood cells (RBCs) (Danesh et al., 2014), as well as tumor cells (Hu et al., 2014; Wen et al., 2016; Gangadaran et al., 2017b; Wang N. et al., 2018). They are detectable in cell culture supernatants (Gangadaran et al., 2018) and human biological fluids (Wang et al., 2018a,b). Exosomes are released to the extracellular space/culture media after fusion of late endosomes/multivesicular bodies (MVBs) with the plasma membrane (Ventimiglia and Alonso, 2016). Exosomes contain an enriched and specific composition of lipids, proteins, mRNAs, miRNAs, and DNAs (Gangadaran et al., 2017a, 2018). Exosomes are crucial players in both local and distant intercellular communications, and play important roles in physiological and pathological processes (Regev-Rudzki et al., 2013; Milane et al., 2015; Devhare et al., 2017). Recently, several reports have demonstrated the use of exosomes as therapeutic agents in anti-cancer therapies (Kalimuthu et al., 2016; Zhu et al., 2017), proangiogenic medicine (Gangadaran et al., 2017c), and regenerative medicine (Rajendran et al., 2017). Furthermore, exosomes have several advantages, including a small size for deep tissue penetration, a slightly negative zeta potential for prolonged blood circulation, and the ability to escape degradation and clearance by the immune system (Hood and Wickline, 2012; Hood, 2016; Vader et al., 2016). In this context, exosomes appear to be attractive candidates for use as exogenous drug delivery vehicles. Recently, several studies have reported that exosomes can carry a considerable amount of exogenous drugs. For example, exosomes are used as nanocarriers for pharmaceutical drugs such as paclitaxel and doxorubicin, both *in vitro* and *in vivo* to inhibit the growth of tumors (Pascucci et al., 2014; Tian et al., 2014). Another study has shown that exosomes are capable of carrying siRNAs and miRNAs to tumor cells and help alter gene expressions which subsequently inhibit proliferation of the cells (Ohno et al., 2013; Banizs et al., 2014).

Despite several studies that have commonly used exosomes as systems to deliver therapeutic agents in the treatment of several diseases, extensive research on exosomes is limited by the extremely small quantity of exosomes produced by cells (Théry et al., 2006). Large-scale production and purification of exosomes is time consuming and expensive (Jang et al., 2013; Oh et al., 2015). However, production of exosome mimetics (EMs) is more feasible compared to exosome production. Therefore, EMs can serve as suitable nanocarriers for drug delivery in translation medicine in the future. As mentioned above, most cells produce exosomes that may have entirely different fates of distribution *in vivo*. Hence, the *in vivo* visualization and tracking of exosomes in animals or humans is crucial for the development of exosomes as drug delivery vehicles that target specific organs or diseases.

In this study, we have developed for the first time, a simple and versatile strategy to produce EMs that can serve as nanocarriers from RBCs by subjecting RBCs to single extrusion through filters with 1 μm pore size, and subsequent purification by density gradients. Finally, the RBC-EMs were labeled with technetium-99m (^99m^Tc), and gamma camera imaging was carried out to visualize and quantitatively evaluate the *in vivo* distribution of the RBC-EMs after intravenous injection into mice.

## Materials and Methods

### Preparation of RBC Exosomes and Exosome-Mimetics

Fresh whole blood was drawn from Sprague-Dawley rats and mixed with the citrate-dextrose anticoagulant solution (Sigma, United States). The RBCs were collected by two centrifugation steps at 1,500 *g* for 20 min at 4°C. RBC-Exosomes (RBC-Exos) were isolated from the RBCs according to a previous protocol (Varga et al., 2016). The RBC-EMs were prepared as described in previous studies (Jang et al., 2013; Oh et al., 2015) with modifications as shown in **Supplementary Figure S1A**. In brief, the RBC concentrate was two-fold diluted in phosphate-buffered saline (PBS). The RBCs were extruded four times through 1 μm-pore sized polycarbonate membrane filters (Nuclepore, Whatman, Inc., Clifton, NJ, United States) using a mini-extruder (Avanti Polar Lipids, Birmingham, AL, United States). Then, the extruded samples were diluted 20-fold with PBS, and centrifuged at 3,000 *g* for 10 min to remove RBCs, larger vesicles, and debris. The centrifuged samples were filtered through a 0.22 μm syringe filter and ultracentrifuged (4°C) at 100,000 *g* for 1 h (Beckman Coulter, Brea, CA, United States). Following this, a two-step iodixanol (OptiPrep, Sigma) density gradient ultracentrifugation (4°C) was performed (Gangadaran et al., 2017c). RBC-EMs were obtained from the crossing point of the 60 and 20% iodixanol layers and used immediately for further experiments. The cumulative amount of proteins in the RBC-Exos and RBC-EMs were calculated as described previously (Gangadaran et al., 2017b).

### Nanoparticle Tracking Analysis (NTA)

The sizes of RBC-Exo, RBC-Ems, and ^99m^Tc**-**RBC-EMs (*n* = 3) were measured by Nano Sight LM 10 (Malver) according to the provided instructions. The samples were diluted 1,000-fold in milli-Q water, and using a sterile syringe, the sample was injected into the chamber and the measurements were made as described previously. The measurements were done in triplicate and analyzed (Gangadaran et al., 2017c).

### ^99m^Tc Labeling of RBC-EMs and Purification

RBC-EMs were incubated with 0.01% Tin (II) chloride (Sigma, United States) in a shaker for 5 min, technetium-99m, ^99m^Tc (RBC-EMs, 100 μg: ^99m^Tc, 111 MBq) was added to the RBC-EMs, and they were further incubated in the shaker for 20 min. Labeling efficiency of RBC-EMs by ^99m^Tc was measured by instant thin-layer chromatography (TLC) using 0.9% NaCl solution as an eluent for each column, and the radioactivity of the column was detected with a radio-TLC imaging scanner (AR-2000, Bioscan, Poway, CA, United States) (Hwang et al., 2015). Stability was determined by the percent change in the radiochemical purity of ^99m^Tc-RBC-EMs over time.

### Measurement of Serum Stability

^99m^Tc-RBC-EMs were incubated in a PBS solution with 20% FBS at 37°C in a CO_2_ incubator (Imai et al., 2015). The stability of ^99m^Tc-RBC-EMs was evaluated using the radio-TLC scanner as mentioned above at 0, 1, 3, and 24 h.

### Transmission Electron Microscopy

Pellets of RBC-Exo, RBC-EMs and ^99m^Tc-RBC-EMs were resuspended in 100 μl of 2% paraformaldehyde. Then, 5 μl of RBC-EMs and ^99m^Tc-RBC-EMs were individually attached to the Formvar-carbon coated with EM grids (Electron Microscopy Sciences, United States) and dried in an open environment for 20 min. Fifty microliters of PBS was placed on a sheet of parafilm, and the upsides of the grids were placed on the drops of PBS using sterile forceps for washing. The grids were then placed onto 50 μl of 1% of glutaraldehyde and incubated at room temperature for 5 min. They were later washed with distilled water for 2 min. The RBC-EMs and ^99m^Tc-RBC-EMs in the grids were stained with 2% uranyl acetate, washed seven times with PBS, allowed to completely dry, and finally observed using a HT 7700 transmission electron microscope (Hitachi, Tokyo, Japan) to measure the size of the EMs (Rajendran et al., 2017).

### Ethics Statement

The study was performed in compliance with the Helsinki Declaration and all the described procedures were reviewed and approved by Kyungpook National University (KNU-2012-43), South Korea.

### *In vivo* Gamma Camera Imaging of ^99m^Tc-RBC-EMs in Mice

*The in vivo* imaging was performed on male C57BL/6 mice (5.5 weeks old) that were obtained from Hamamatsu (Shizuoka). The gamma camera images were captured for 10 min using a 2 mm pinhole collimator (Infinia II, GE Healthcare, Milwaukee, WI, United States). 37 MBq of ^99m^Tc-RBC-EMs in 200 μl volume was injected into the tail vein of the mice (*n* = 4). Control mice (*n* = 4) were injected with same quantity of free ^99m^Tc. During imaging, the mice were kept anesthetized using 2.5% isoflurane (Merial, Lyon, France). The gamma camera images were taken at 1 and 3 h post-administration of ^99m^Tc-RBC-EMs or free ^99m^Tc. Region of interests (ROIs) were drawn on thyroid gland and stomach in free-^99m^Tc injected mice, thyroid and liver/spleen in ^99m^Tc-RBC-EMs injected mice. And right thigh area was considered as control ROIs. Average counts per pixel of ROIs were divided by control ROI in same mice.

### Biodistribution of ^99m^Tc-RBC-EMs in Mice

^99m^Tc-RBC-EMs or free ^99m^Tc were intravenously injected into mice (*n* = 4/group). One hour later, the mice were sacrificed after drawing blood samples from all mice. The uptake values were measured in the following organs: lungs, heart, liver, stomach, spleen, intestine, kidney, muscle, and thyroid using a gamma-counter (Cobra II) (Oh et al., 2017). The data were expressed as the injected dose per gram tissue in percentage (%ID/g).

### *In vivo* and *ex vivo* Fluorescent Imaging of RBC-EM ^DiD^ in Mice

RBC-EMs were incubated with 1,1’-Dioctadecyl-3,3,3’,3’-Tetramethylindodicarbocyanine Perchlorate (DiD; Thermo Fisher Scientific) for 20 min at room temperature, washed with PBS, and a two-step Opti-Prep density gradient ultracentrifugation was performed as described above. The C57BL/6 mice were anesthetized using 2.5% isoflurane (Merial) and RBC-EM ^DiD^ (*n* = 3) or PBS (*n* = 3) was intravenously injected through their tail vein. FLI was performed using an *in vivo* imaging system (IVIS Lumina III instrument, PerkinElmer) at 1 and 60 min post-injection. [wavelengths: Excitation – 644 nm and emission – 665 nm; imaging parameters: binning – 4, smoothing – 3 × 3, Field of subject (stage – D): 12.5 cm, height of subject image: 1.5 cm] After the imaging, the liver and spleen of the mice were harvested, *ex vivo* FLI was performed, and quantification was done using IVIS software (Living Image Software, PerkinElmer).

### Immunofluorescent Assay

Liver tissues were cryo-sectioned and processed for the immunofluorescence (IF) assay as described previously (Alam et al., 2012). RBC-EM ^DiD^ or PBS-injected mouse liver sections were stained with anti-rabbit CD68 (Abcam), followed by staining with goat anti-rabbit FITC (Abcam). Tissue sections were mounted using the VECTASHIELD mounting medium (Vector Laboratories, Burlingame, CA, United States). IF-stained sections were imaged under a confocal microscope (LSM 5 Exciter, Zeiss, Oberkochen, Germany). Total of six fields were counted by observers. Number of CD68 positive (CD68 +; green) cells were counted from RBC-EM ^DiD^ or PBS-injected mouse liver sections. Then the number of DiD positive (DiD +; red) with CD68 + or CD68 - cells were counted.

### Statistical Analyses

Data are presented as mean ± standard deviation (SD). Statistical significance was determined (student *t*-test) by GraphPad Prism7 software version 7.04 (GraphPad Software, Inc., La Jolla, CA, United States). *P-*values less than 0.05 were considered statistically significant.

## Results

### Production and Characterization of RBC-EMs and RBC-Exos

**FIGURE 1:**
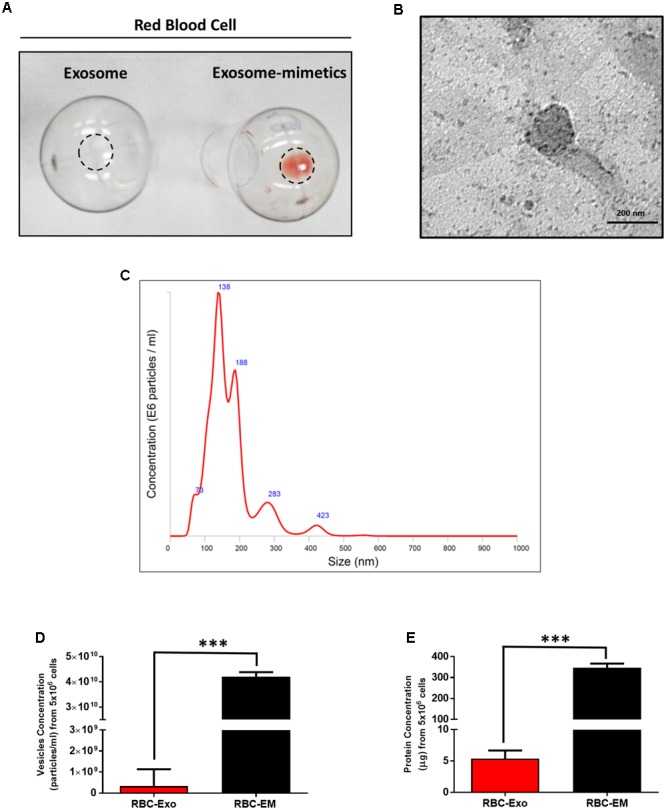
Characterization of RBC-EMs and RBC-Exos, and comparison of their vesicular yields **(A)** Representative images of RBC-Exos and RBC-EMs isolated from the same number of RBCs. **(B)** Morphology of RBC-EMs and ^99m^Tc-RBC-EMs was examined by TEM. **(C)** Nanoparticle tracking analysis of RBC-Exos. **(D)** The yields of RBC-Exos and RBC-EMs measured as particle numbers (*n* = 3). **(E)** RBC-Exos and RBC-EMs total protein content (*n* = 3). The values are expressed as mean ± SD, ^∗∗∗^*P* < 0.001, (Student’s *t*-test).

For the large-scale generation of EMs, rat RBCs were isolated from whole blood, extruded through a series of polycarbonate membranes of 1 μm pore size, and purified (removal of membrane fragments, free hemoglobin) from the interface layer through Opti-Prep density gradient (**Supplementary Figures S1A–C**). These EMs were termed as RBC-EMs. To compare the production of natural RBC-Exos to RBC-EMs, exosomes were isolated by the gold standard methods of ultracentrifugation (**Supplementary Figure S1D**). The representative visual images of RBC-Exos and RBC-EMs are shown in **Figure 1A**, which were isolated or generated from the same number of RBCs. Transmission electron microscopic analysis showed a classical round shape morphology of RBC-Exo (**Figure 1B**). The NTA analysis revealed that the mean size of RBC-Exos was 209.1 ± 19.8 nm and ranged between 30 and 450 nm (**Figure 1C**). Further, we determined the number of RBC-Exos and RBC-EMs produced with 5 × 10^6^ RBCs by conventional ultracentrifugation and extraction, respectively. Significantly higher numbers (∼130-fold) of RBC-EMs were obtained compared to RBC-Exos from the same number of RBCs (*P* < 0.001, **Figure 1D**). Next, we determined the protein concentration of RBC-EMs and RBC-Exos. Similar to the results of the vesicle numbers, RBC-EMs showed significantly higher protein concentration (∼65-fold) than RBC-Exos (*P* < 0.001, **Figure 1E**). Taken together, these results demonstrated the successful generation of RBC-EMs on a large-scale compared to the conventional generation of natural RBC-Exos from RBCs.

### Radiolabeling of RBC-EMs With ^99m^Tc

**FIGURE 2:**
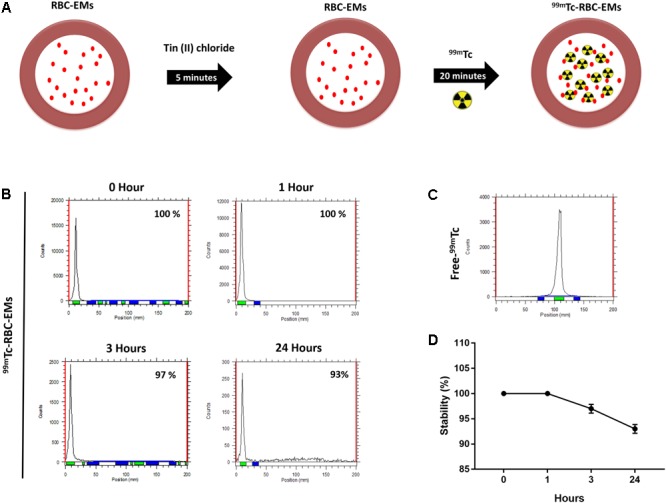
Radiochemical purity and stability of ^99m^Tc-labeled RBC-EMs **(A)** Illustration of the procedure for labeling RBC-EMs with ^99m^Tc. **(B,C)** The counts of radioactivity for ^99m^Tc and ^99m^Tc-RBC-EMs were measured by thin layered chromatography. **(D)** Serum stability was determined by the percent change in the radiochemical purity of ^99m^Tc-RBC-EMs for the indicated time periods. The values are expressed as mean ± SD.

The RBC-EMs were incubated with Tin (II) chloride followed by ^99m^Tc-pertechnetate for labeling as showed in **Figure 2A**. The radiochemical purity was 100% after purification with centrifugation at 0 h (**Figure 2B**), with free ^99m^Tc used as a control (**Figure 2C**). The stability of ^99m^Tc-RBC-EMs in serum was tested. The percentage of ^99m^Tc-RBC-EMs in serum was 100% (1 h), 97 ± 2.5% (3 h), and 93 ± 3% (24 h) (**Figures 2B,D**). As noted above, RBC-EMs were successfully labeled with ^99m^Tc. These ^99m^Tc-RBC-EMs were used for further characterization, gamma camera imaging, and analyzing *ex vivo* biodistribution in living animals.

### Characterization of RBC-EMs after ^99m^Tc Radiolabeling

**FIGURE 3:**
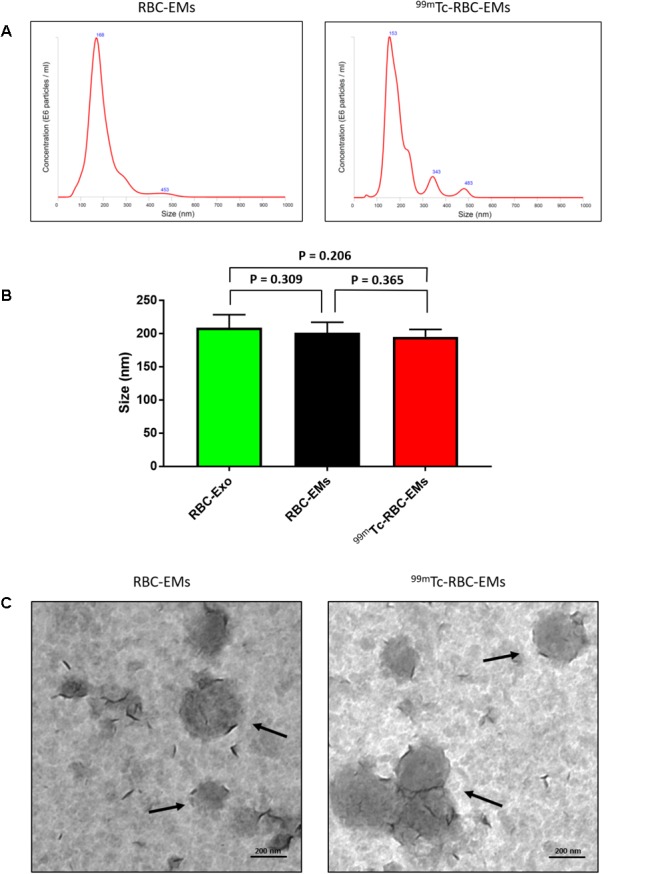
Typical characteristics of RBC-EMs and ^99m^Tc-RBC-EMs. **(A)** Average size distribution of RBC-EMs and ^99m^Tc-RBC-EMs as examined by NTA. **(B)** Average size of RBC-Exo, RBC-EMs and ^99m^Tc-RBC-EMs were represented in bar graph (*n* = 3) and there was no significant (NS) difference (*P* > 0.05) in the size distribution between amount the all three groups. **(C)** Morphology of RBC-EMs and ^99m^Tc-RBC-EMs was examined by TEM. There were no visible differences in the morphology between RBC-EMs and ^99m^Tc-RBC-EMs. The values are expressed as mean ± SD (Student’s *t*-test).

We performed nanoparticle analysis and transmission electron microscopic analysis to test whether the ^99m^Tc labeling procedure affected the size and morphology of the RBC-EMs. NTA results showed that the average diameter of RBC-EMs and ^99m^Tc-RBC-EMs were 201.3 ± 16 and 195.0 ± 11.1 nm, respectively (**Figures 3A,B**), which confirms that there was no significant (RBC-EMs Vs ^99m^Tc-RBC-EMs: *P* = 0.365) change in size after ^99m^Tc labeling to RBC-EMs and RBC-Exo (RBC-Exo Vs RBC-EMs: *P* = 0.309; RBC-Exo Vs ^99m^Tc-RBC-EMs: *P* = 0.206) (**Figure 3B**). The scattered diagram of RBC-Exo, RBC-Ems, and ^99m^Tc-RBC-EMs were represented in **Supplementary Figure S2**. TEM was performed to confirm whether the radio-labeling changed the shape or morphology of the RBC-EMs. Our results showed that no changes were observed by TEM. Both the unlabeled and labeled RBC-EMs showed a similar round shape and no damages to EMs were observed (**Figure 3C**). These results indicate that the radio-labeling does not alter the size and shape of RBC-EMs.

### *In vivo* Gamma Camera Imaging and Biodistribution of ^99m^Tc-RBC-EMs

**FIGURE 4:**
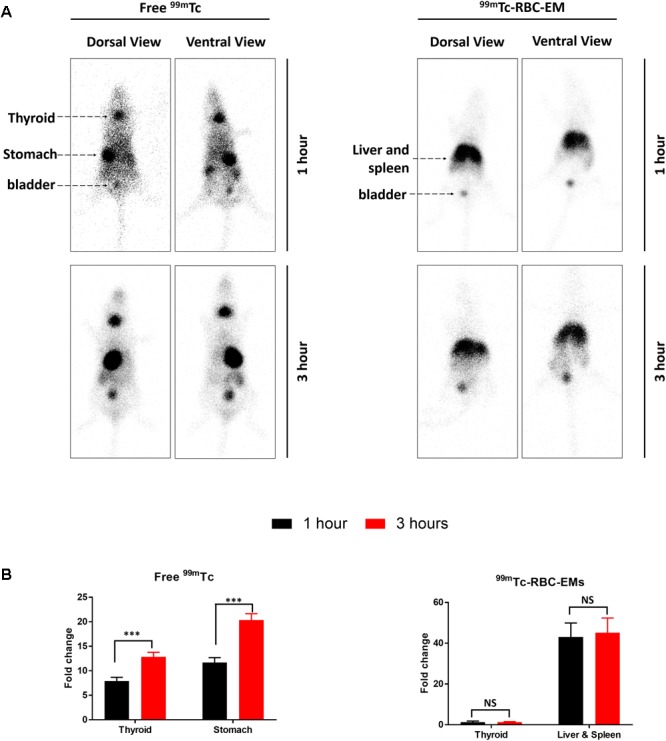
*In vivo* gamma camera images of free ^99m^Tc and ^99m^Tc-RBC-EMs injected in mice. **(A)** After the intravenous injection of free ^99m^Tc and ^99m^Tc-RBC-EMs, gamma camera images were acquired at 1 and 3 h post-injection in C57BL/6 mice (*n* = 4). **(B)** Quantification of ^99m^Tc and ^99m^Tc-RBC-EMs injected mice organ (thyroid, stomach, liver, and spleen) was performed and represented as bar graph. The values are expressed as mean ± SD, ^∗∗∗^*P* < 0.001 (Student’s *t*-test).

**FIGURE 5:**
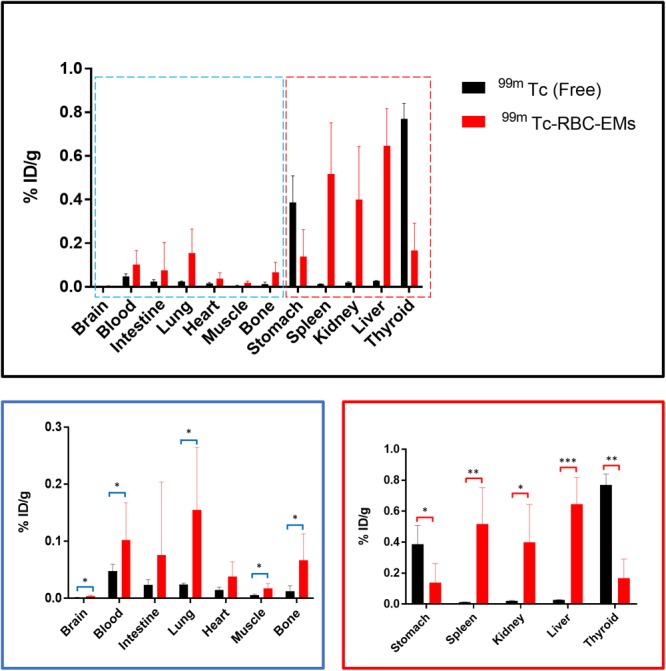
Biodistribution of free ^99m^Tc and ^99m^Tc-RBC-EMs. Mice were intravenously injected with free ^99m^Tc and ^99m^Tc-RBC-EMs. After 1 h, the mice were sacrificed and the radioactivity in the dissected organs was measured. Data are expressed as injected dose per gram as percentage (%ID/g). The values are expressed as mean ± SD, ^∗^*P* < 0.05, ^∗∗^*P* < 0.01, ^∗∗∗^*P* < 0.001, (Student’s *t*-test).

After the generation and isolation of ^99m^Tc-RBC-EMs, and intravenous injection in mice, we obtained *in vivo* gamma camera images of the ^99m^Tc-RBC-EMs. The *in vivo* gamma camera images revealed that the ^99m^Tc-RBC-EMs were clearly detectable in the living mice. Images acquired 1 h after injection of free ^99m^Tc and ^99m^Tc-RBC-EMs showed the intense uptake of free ^99m^Tc in the thyroid, stomach, and bladder (upper left panel, **Figure 4A**). At the same time, ^99m^Tc-RBC-EMs uptake was seen in the liver, spleen, and bladder (upper right panel, **Figure 4A**). In contrast to free ^99m^Tc, thyroidal or gastric uptake of ^99m^Tc-RBC-EMs was not visualized in mice. Further, at 3 h post-injection, gamma camera images showed a similar pattern as 1 h images in mice injected with free ^99m^Tc and ^99m^Tc-RBC-EMs (lower right and left panel, **Figure 4A**). but free ^99m^Tc showed significantly (*P* < 0.001) increased thyroidal and stomach uptake in 3 h compare to 1 h (left panel **Figure 4B**); ^99m^Tc-RBC-EMs showed no significant (*P* > 0.05) increased thyroidal and liver and spleen uptake in 3 h compare to 1 h (right panel **Figure 4B**). Next, free ^99m^Tc and ^99m^Tc-RBC-EMs were administered to mice via intravenous injection and their biodistribution was assessed. Thyroid uptake of the ^99m^Tc-RBC-EMs was significantly (*P* < 0.01) lower compared to that of free ^99m^Tc. A similar uptake trend was observed in the stomach (*P* < 0.05) as well. Uptake of ^99m^Tc-RBC-EMs in other organs [liver (*P* < 0.001), spleen (*P* < 0.01), and kidneys (*P* < 0.05)] was ∼20–40-fold higher compared to the free ^99m^Tc uptake in these organs. Furthermore, the brain (*P* < 0.05), blood (*P* < 0.05), lungs (*P* < 0.05), muscle (*P* < 0.05), and bone (*P* < 0.05) also showed significantly higher ^99m^Tc-RBC-EMs than free ^99m^Tc (**Figure 5**). These results indicate that the radio-labeling could be used to label EMs, and that the ^99m^Tc-RBC-EMs can be well visualized in living animals.

### *In vivo* Fluorescent Imaging and Sub Cellular Visualization of RBC-EMs ^DiD^

**FIGURE 6:**
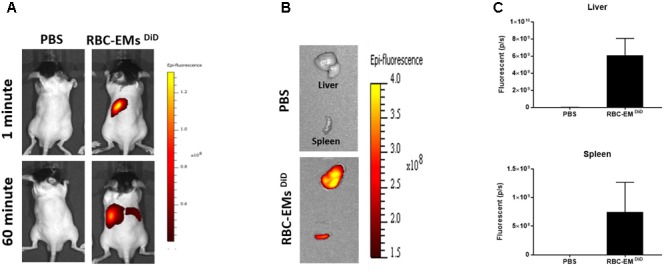
*In vivo* fluorescent imaging of RBC-EMs^DiD^ injected in mice. **(A)** Representative *in vivo* fluorescent imaging of RBC-EMs^DiD^ in mice. RBC-EMs^DiD^ or PBS (control) was administered via the tail vein (*n* = 3). **(B,C)** Representation and quantification of RBC-EMs^DiD^ or PBS (control) signal from the liver and spleen 1 h after EMs administration; the values are expressed as mean ± SD.

**FIGURE 7:**
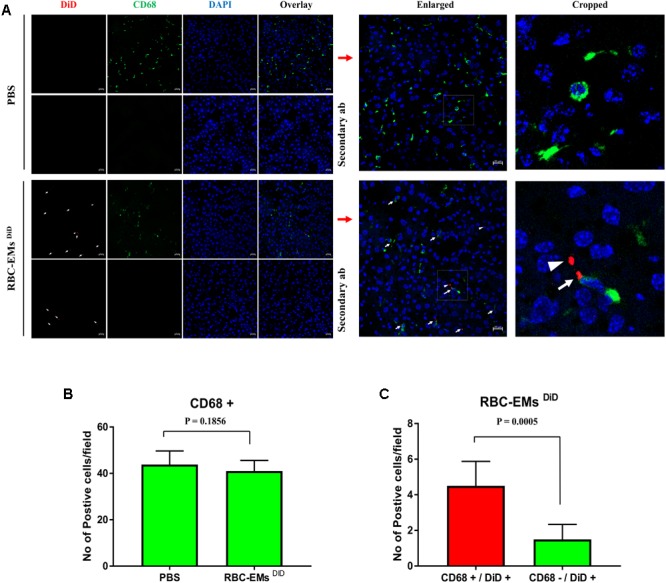
Sub-cellular visualization of RBC-EMs DiD in mice liver. **(A)** Representative confocal images of RBC-EMs^DiD^ (red) and CD68 (green) in liver harvested from the mouse mentioned in. Arrow indicating CD68 + and DiD +; arrow head indicating CD68 – and DiD +Scale bars: 20 μm. **(B)** Quantification of CD68 cells from confocal images of liver from PBS and RBC-EMs^DiD^ injected mice was counted and represented in bar graph. **(C)** Quantification of confocal images of RBC-EMs^DiD^ (red) with or without CD68 (green) in liver harvested from the mouse was counted and represented in bar graph. The values are expressed as mean ± SD (Student’s *t*-test was used).

To investigate the biodistribution of the RBC-EMs, they were labeled with DiD (**Supplementary Figures S3A,B**), and then intravenously administered to the mice. Fluorescence imaging immediately after injection showed strong signals in the region of liver, and 60 min post-injection images showed stronger signals at the region of the liver and spleen. Whereas, control (PBS) mice did not show any signals (**Figure 6A**). Fluorescent imaging confirmed the RBC-EMs ^DiD^ were significantly distributed to liver and spleen *ex vivo* (**Figures 6B,C**). We further analyzed the localization of the RBC-EMs. First our results revealed that no significant (*P* = 0.1856) number of CD68 positive cells (kupffer cells – liver resident macrophage cells) present in the liver sections (**Figures 7A,B**). Further our immunofluorescent analysis revealed that significant number (*P* < 0.0005) of the RBC-EMs^DiD^ were co-localized with kupffer cells; which is about 75% of RBC-EMs^DiD^ were localized to kupffer cells and about 25% of RBC-EMs^DiD^ were not localized to kupffer cells (**Figures 7A,C**). CD68 + cells in control mice showed no DiD signal (**Figures 7A,C**). Taken together, these results indicate that RBC-EMs were distributed predominantly to the liver and were internalized into Kupffer cells (macrophage) cells.

## Discussion

A safe drug or nucleic acid delivery system to target specific tissues in the body is crucial to accomplish the desired therapeutic effect and control the disease without any side-effects. RBC, pancreatic cells, platelets and stem cell, have been studied in cell-based therapy due to their long circulation and specific tropism to diseased tissue. (Ye et al., 2016; Wang et al., 2017a,b; Kalimuthu et al., 2018). Recently, drug delivery by exosomes receives a great deal of attention due to its advantages as naturally obtained nanocarriers that can avoid several hurdles, including off-target effects, unwanted immune activation, and most importantly *in vivo* degradation, making exosomes favorable drug delivery vehicles (Gangadaran et al., 2018) with therapeutic abilities (Suntres et al., 2013).

For the successful use of exosomes as drug delivery vehicles in research and clinical translation, large-scale production of exosomes and *in vivo* tracking of exosomes are essential. EMs have similar characteristics as that of exosomes, but have a 100-fold higher production yield (Jang et al., 2013). It is important to note that crucial components of exosomes are still largely unknown, and the integrative studies are required to elucidate the biological functions of these components. Furthermore, EMs have been used to deliver chemotherapeutic drugs to inhibit tumors and RNAi to attenuate the target gene expression (Jang et al., 2013; Lunavat et al., 2016).

In this study, we used RBCs for the production of EMs because the collection of blood from humans is easier and less-invasive compared to isolation of the most frequently used mesenchymal stem cell exosomes for exosome-based therapies/drug delivery (Lai et al., 2013; Kalimuthu et al., 2016; Lou et al., 2017). If the patients’ own RBC-EMs are used as drug delivery vehicles, any graft versus host reactions can be prevented. Further studies are needed to uncover the mechanisms of the interaction of RBC-EMs among different species. In this study, we produced EMs from RBCs by the extrusion method using single membrane filters and compared them with the conventional exosomes, RBC-Exos. The RBC-EMs did not show significant differences in the morphology, size, and range of distribution, which agree with previous studies (Jang et al., 2013; Hwang et al., 2015). Furthermore, we produced more than 130-fold higher RBC-EMs with more than 60-fold higher total protein compared to the naturally produced RBC-Exos, respectively. Other previous studies also showed a similar trend (Hwang et al., 2015; Lunavat et al., 2016). Recent study reported that RBC exosomes are formed and released only during the early developmental stage of RBCs in bone marrow (Kuo et al., 2017) as maturing RBC eject their nuclei to contain more oxygen-carrying hemoglobin (Ji et al., 2008). Exosome biogenesis is complex and step-by-step process (Gangadaran et al., 2018) can be executed by proliferating cells with nucleus but RBC doesn’t possesses a nuclease to perform such a complex work. RBCs shown to only releases extracellular vesicles during the aging by budding (Kuo et al., 2017). Hence, we solved a major issue faced by exosome research groups by improving the productivity of EMs, using the same number of RBCs from which conventional methods can only generate an extremely small number of exosomes.

In this study, we employed radio-labeling of RBC-EMs to apply real time *in vivo* nuclear imaging in living animals. Visualization and tracking of the exosomes were mostly carried out with BLI or FLI by transducing the cells with reporter genes (Takahashi et al., 2013; Lai et al., 2014; Kalimuthu et al., 2016; Gangadaran et al., 2017b) or with FLI by labeling the cells with lipophilic dyes (Mittelbrunn et al., 2011; Gangadaran et al., 2017b). However, optical imaging suffers from low spatial resolution and tissue penetration and is still not suitable for clinical translation (Tavri et al., 2009; Gangadaran and Ahn, 2017). Nuclear imaging provides excellent sensitivity and good tissue penetration (Ahn, 2014). To our knowledge, there are only a few studies that have reported the development of radio-labeled exosomes to assess biodistribution. However, three out of those reports, showed only the *ex vivo* biodistribution (Morishita et al., 2015; Smyth et al., 2015; Varga et al., 2016), and one study used streptavidin reporters which need genetically modified cells (Morishita et al., 2015).

When we generated and purified RBC-EMs, the purified pellets were red colored due to the presence of hemoglobin. ^99m^Tc-labeled RBCs are widely used for detecting the bleeding focus in humans (Dam et al., 2014), and tin (II) chloride is used as a reducing agent. ^99m^Tc is changed by tin (II) chloride to a lower oxidation state that firmly binds to hemoglobin. The labeling efficiency of ^99m^Tc to RBC is more than 95%. In this study, we labeled ^99m^Tc-pertechnetate to hemoglobin inside the RBC-EMs. ^99m^Tc labeling resulted in excellent radiochemical purity (almost 100%). Furthermore, RBC-EMs retained ∼97% of the entrapped ^99m^Tc under serum conditions for up to 3 h. Even after 24 h, the radiochemical purity was ∼93%.

When macrophage- or stem cell-derived extracellular nanovesicles are labeled with ^99m^Tc-HMPAO, the labeling efficiency is usually so low that further purification procedures are needed. The radiochemical purity of ^99m^Tc-HMPAO was 99.6% (Elution over PD-10 Column) and 93.7% (Centrifugation with exosome exclusive spin column) after purification. These column purification methods may cause loss of some exosomes in the process (Hwang et al., 2015). In contrast, we didn’t use any column method for purification as the initial radiochemical purity was 100% (purity remained the same up to 2 h). Radiolabeling of RBC extracellular vesicles was recently reported using the ^99m^Tc-tricarbonyl method. Their labeling efficiency was approximately only 38% (Varga et al., 2016), which could be reasonable for small animal studies but not for clinical studies, and may not be good for tracking high concentrations of exosome injection studies. The previous study mentioned that ^68^Ga labeling using a bifunctional chelator can lead to aggregation, which results in its accumulation in the lungs (Hwang et al., 2015). To avoid such issues, we checked whether our labeling method changed the size and morphology of exosomes. We found no significant changes in the size distribution of unlabeled RBC-EMs and ^99m^Tc labeled RBC-EMs. Furthermore, we also found no changes in the morphology of RBC-EMs due to the labeling.

Non-invasive *in vivo* gamma camera imaging of ^99m^Tc-RBC-EMs in living mice revealed that it is very sensitive and quantitative. Imaging of intravenously injected ^99m^Tc-RBC-EMs in mice showed that most of the RBC-EMs accumulated in the liver and spleen, followed by the bladder, at 1 and 3 h post-injection. Whereas, free ^99m^Tc-administrated mice showed significant uptake in the thyroid, stomach, and bladder. There was no uptake of ^99m^Tc-RBC-EMs in the thyroid of injected mice, which clearly showed that labeling was stable not only *in vitro* also *in vivo*. The accumulation of ^99m^Tc-RBC-EMs in the liver and spleen could be a result of phagocytosis of the RBC-EMs by the mononuclear phagocyte system (Mirahmadi et al., 2010) and our results are consistent with the previous studies (Hwang et al., 2015; Varga et al., 2016; Gangadaran et al., 2017b). Our approach has demonstrated that the nuclear imaging employed provides excellent sensitivity and enables the visualization of RBC-EMs in deep organs such as the liver and spleen. Nuclear imaging using radionuclides has been widely and readily used for clinical purposes. Additionally, our *ex vivo* quantification in mice organs further confirmed the uptake of free ^99m^Tc and ^99m^Tc-RBC-EMs. Most of the ^99m^Tc-RBC-EMs were accumulated in the liver (24-fold), spleen (42-fold), and kidneys (20-fold) compared to those in free ^99m^Tc-administered mice at 1 h. Thyroid uptake was fivefold higher with free ^99m^Tc administration compared to ^99m^Tc-RBC-EMs administration. Furthermore, the lungs, bones, and heart also showed ∼2–7-fold higher ^99m^Tc-RBC-EMs accumulations than that of free ^99m^Tc. This differential distribution confirmed that ^99m^Tc labeling of RBC-EMs is stable in mice.

Further, we verified the *in vivo* biodistribution of RBC-EMs by dye-based direct labeling. The fluorescent signal (RBC-EMs ^DiD^) was first observed in the region of the liver immediately after administration and at 1 h post-administration. Further, immunofluorescent assay showed sub cellular visualization of RBC-EMs ^DiD^ in liver. Most of the RBC-EMs ^DiD^ were co-localized with the CD68 positive kupffer cells. This is consistent with the previous report (Gangadaran et al., 2017b). As RBC-EMs are derived from RBCs, they potentially have advantages in preclinical and clinical uses for imaging and as drug carriers as well. Several studies have exploited exosomes as drug carriers for the treatment of several diseases (Jang et al., 2013; Banizs et al., 2014; Pascucci et al., 2014; Tian et al., 2014; Lunavat et al., 2016; Gangadaran et al., 2018). Our approach of producing EMs from RBCs is simple and it would pave the way for its large-scale production. RBC-EMs can be used to load drugs and deliver the loaded drug to Kupffer cells in the liver. Radiolabeled RBC-EMs can be used to track *in vivo* drug delivery in preclinical and clinical scenarios.

The most studied drug delivery platform are liposomes and polymeric nanoparticles, both nano-carriers have been used to deliver drug *in vivo* (Gangadaran et al., 2018). Recently exosomes have been investigated more in exosome-based therapies (Kalimuthu et al., 2016; Zhu et al., 2017, 2018) and drug deliveries (Tian et al., 2014; Gangadaran et al., 2018). RBC-EMs distributed to liver and spleen rapidly within 1 h, this character of RBC-EMs can be utilized in diseases such as hepatic fibrosis to target liver resident macrophages as macrophages are real mediator of these liver diseases (Tacke, 2017). RBC-EMs shown to have short-circulation time, which can be changed by various methodologies such as, a blocking of scavenger receptor class A (SR-A) as a monocyte/macrophage uptake receptor for exosomes. *In vivo* blockade of SR-A with dextran sulfate dramatically decreased exosome liver clearance in mice, while enhancing tumor accumulation (Watson et al., 2016; Zhu et al., 2018); macrophage-depletion by clodronate in mice shown to improve the longer circulation time and slower clearance *in vivo* (Imai et al., 2015); Recently, tumor-targeting peptides have been used to target tumors with cytotoxic T cells or anti-apoptotic peptides to inhibit the tumor *in vivo* (Sarangthem et al., 2016; Gunassekaran et al., 2018), and another report has shown that exosomes labeled with cardiac homing peptides can be used to target myocardial infarction *in vivo* (Vandergriff et al., 2018). Such a targeting approach can be used to direct the RBC-EMs to the target site with therapeutic drugs loaded in them, and the RBC-EMs containing therapeutic drugs can be feasibly monitored *in vivo* by ^99m^Tc labeling which can accelerate development of the drug delivery system in clinics. Since RBC-EMs can be produced immediately after collecting the bloods from patient and injected into same patient, which should be a highly biocompatible compared to other drug carriers. In addition, specific exosome (NK exosome, macrophage-exosome) shown to have intrinsic properties to target, kill tumors and modulate inflammation *in vivo* (Yuan et al., 2017; Zhu et al., 2018). Further studies are needing to compare the utilization of RBC-EMs as an additional drug delivery carrier.

In summary, we have developed a robust and simple method for the ^99m^Tc labeling of RBC-EMs. This labeling procedure was stable both *in vitro* and *in vivo*. The non-invasive nuclear imaging of ^99m^Tc-cRBC-EMs is a promising approach to investigate the *in vivo* behavior of RBC-EMs and can used for developing RBC-EM-based drug delivery systems.

## Author Contributions

PG and B-CA initiated the project. PG, CH, JO, and RR conducted most of the experiments and data analysis. SK, SS, AG, LZ, and SB conducted some of the experiments. SJ, S-WL, JL, and B-CA provided the tools, materials, and methodologies. PG prepared the manuscript. All authors approved the final version of the manuscript.

## Conflict of Interest Statement

The authors declare that the research was conducted in the absence of any commercial or financial relationships that could be construed as a potential conflict of interest.

## Supplementary Material

FIGURE S1 Schematic diagram of the generation and purification of RBC-exosome mimetics and the isolation of RBC-exosomes. **(A)** Schematic illustration of the procedure for the generation of exosome mimetics. **(B)** Schematic illustration of extrusion. **(C)** Representative images of purification of RBC-EMs by Opti-prep density gradient ultracentrifugation. **(D)** Schematic illustration of the procedure for the isolation of exosomes.

FIGURE S2 Scattering images obtained during NTA of RBC-Exo, RBC-EMs, ^99m^Tc-RBC-EMs (*n* = 3).

FIGURE S3 Labeling of RBC-EMs with fluorescent imaging dye (DiD). **(A)** Representative images of unlabeled- RBC-EMs and DiD-labeled-RBC-EMs. **(B)** Quantitation of RBC-EMs and RBC-EMs^DiD^ of **A**. The values are expressed as mean ± SD.
