# Supplementary figures and images for: In vivo Non-invasive Imaging of Radio-Labeled Exosome-Mimetics Derived From Red Blood Cells in Mice
Source: Front Pharmacol. 2018 Jul 30;9:817. doi: 10.3389/fphar.2018.00817 (PMC6078013; doi:10.3389/fphar.2018.00817)

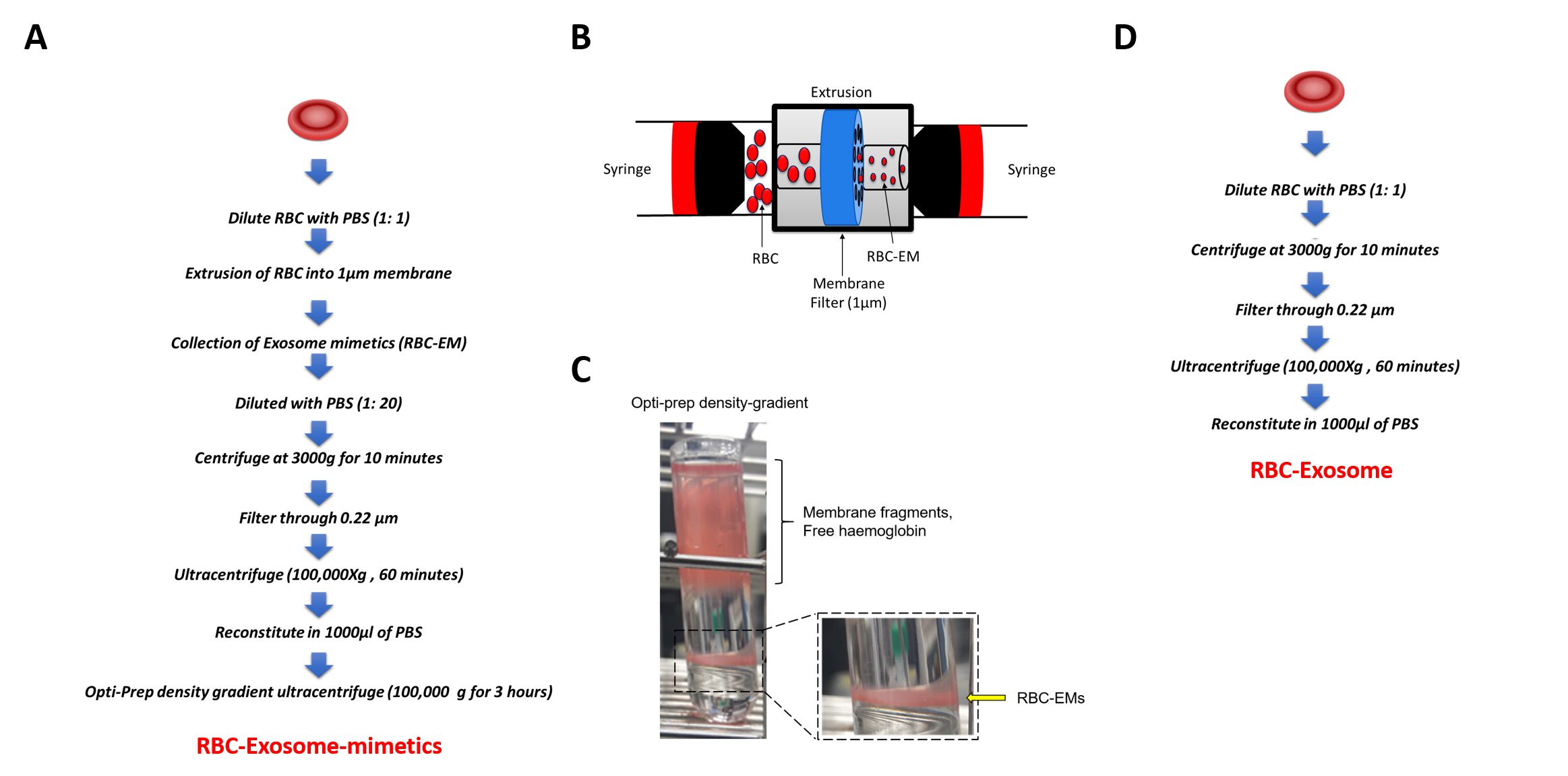

Supplement: FIGURE S1 — Schematic diagram of the generation and purification of RBC-exosome mimetics and the isolation of RBC-exosomes. (A) Schematic illustration of the procedure for the generation of exosome mimetics. (B) Schematic illustration of extrusion. (C) Representative images of purification of RBC-EMs by Opti-prep density gradient ultracentrifugation. (D) Schematic illustration of the procedure for the isolation of exosomes. [file Image_1.TIF]

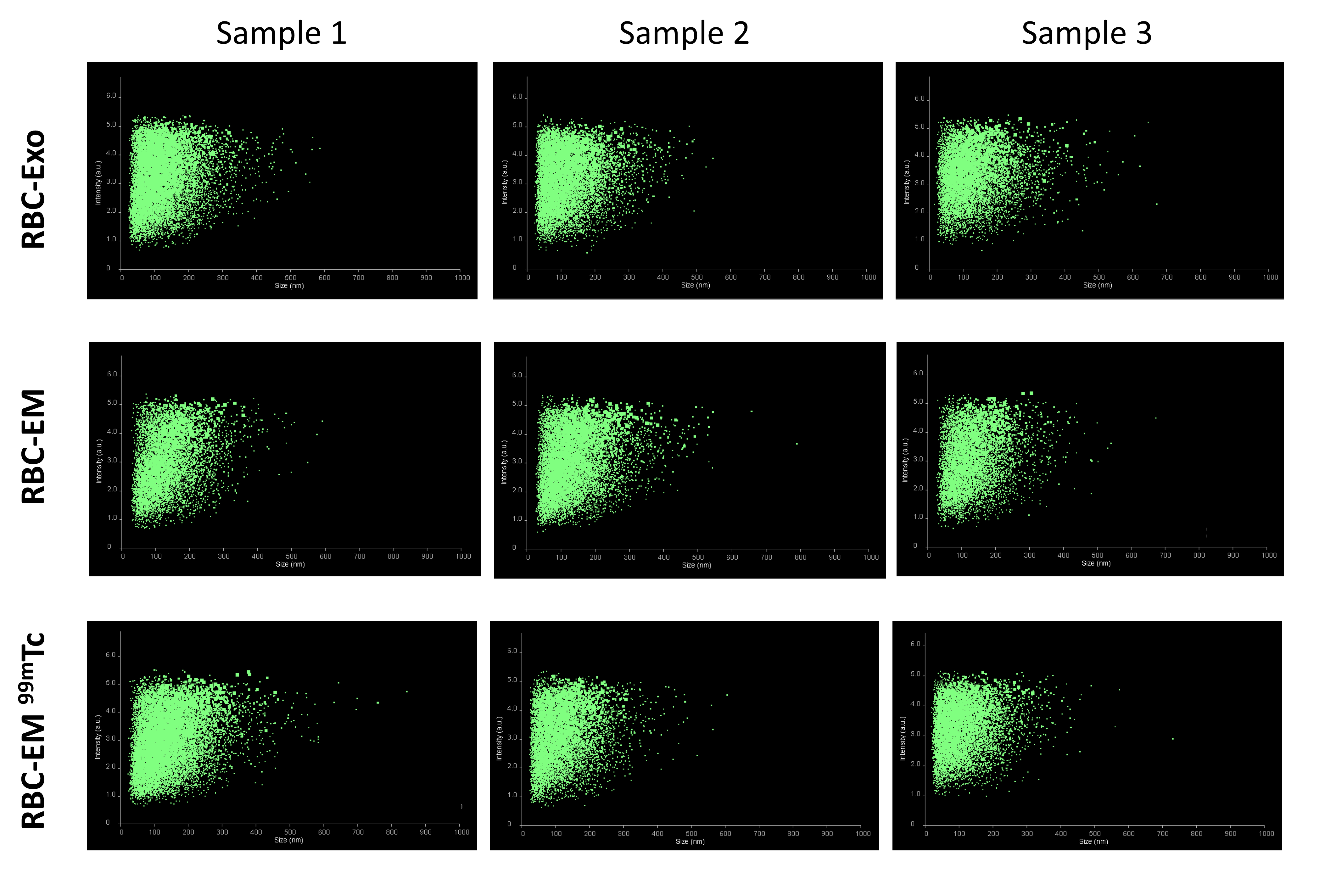

Supplement: FIGURE S2 — Scattering images obtained during NTA of RBC-Exo, RBC-EMs, 99mTc-RBC-EMs (n = 3). [file Image_2.TIF]

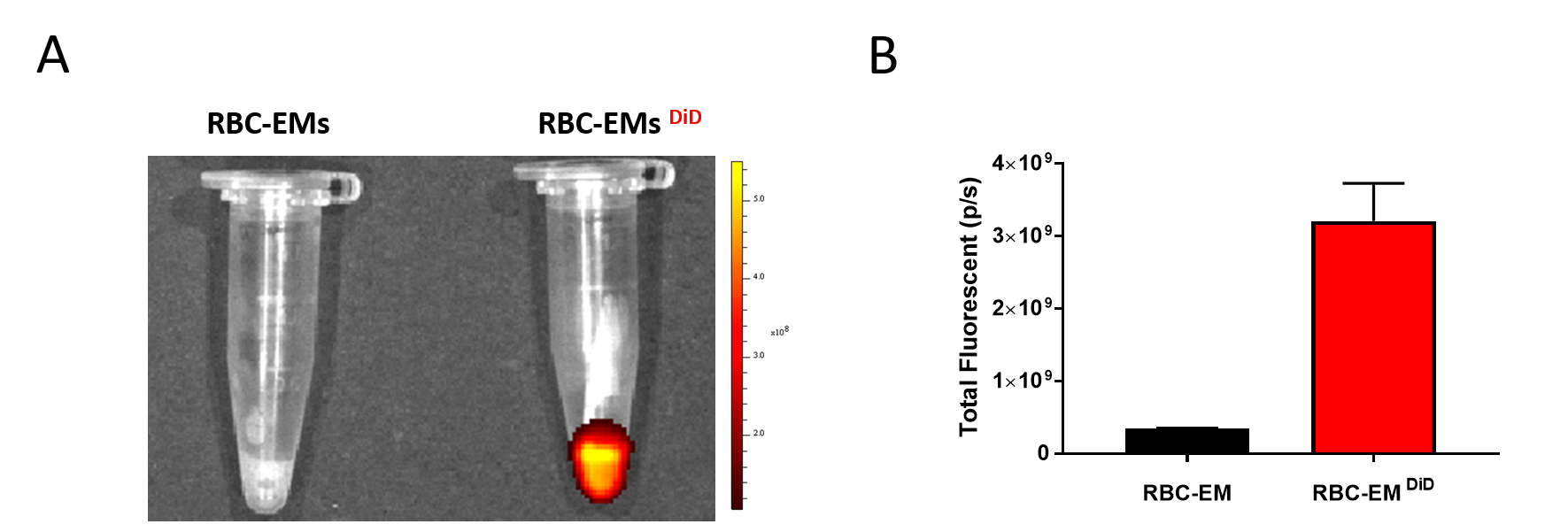

Supplement: FIGURE S3 — Labeling of RBC-EMs with fluorescent imaging dye (DiD). (A) Representative images of unlabeled- RBC-EMs and DiD-labeled-RBC-EMs. (B) Quantitation of RBC-EMs and RBC-EMsDiD of A. The values are expressed as mean ± SD. [file Image_3.TIF]
